# Managing Asymmetry in Breast Reconstruction After Mastectomy—A Systematic Review and Highlight of Clinical Pearls

**DOI:** 10.3390/jcm13237189

**Published:** 2024-11-27

**Authors:** Kelsey Lipman, Dung Nguyen

**Affiliations:** Division of Plastic and Reconstructive Surgery, Stanford University, Stanford, CA 94305, USA; klipman@stanford.edu

**Keywords:** breast reconstruction, breast asymmetry, secondary procedures, revision procedures

## Abstract

**Background/Objectives:** As breast reconstruction techniques continue to progress, patient satisfaction with aesthetic outcomes has become an increasingly important marker of success. Obtaining optimal symmetry often requires secondary procedures whether reconstruction is unilateral or bilateral, implant-based or autologous, immediate or delayed. Consequently, determining the ideal method to achieve symmetry, particularly in challenging scenarios, such as the radiated breast, is nuanced and requires experienced decision-making. **Methods:** A systematic review was performed according to the Preferred Reporting Items for Systematic Reviews and Meta-Analyses (PRISMA) statement guidelines, including the PubMed, Cochrane Library, and Embase bibliographic databases, to identify original articles addressing asymmetry in both implant-based and autologous breast reconstruction. Studies based on benign breast disease or oncoplastic reconstruction for partial mastectomy/lumpectomy defects were excluded. **Results:** The search initially yielded a total of six hundred and fifty unique articles. After complete assessment of inclusion and exclusion criteria, a total of forty-one articles were included in total. **Conclusions:** This article provides a systematic review of the current literature available to guide surgeons on managing asymmetry in breast reconstruction and highlights case examples of frequently encountered clinical challenges. A novel treatment algorithm was then generated to serve as a comprehensive decision-making guide for both patients and surgeons.

## 1. Introduction

Patient satisfaction after breast reconstruction is becoming an increasingly important postoperative marker of success, with symmetry between breasts playing a key role. Though symmetry can sometimes be obtained with a single-stage operation, secondary procedures are often necessary to achieve optimal results. These secondary procedures may be necessary both in implant-based methods or autologous reconstruction, whether the reconstruction is immediate or delayed, and in the setting of both unilateral and bilateral procedures. As a result, determining the best method to address asymmetry is nuanced and can be a challenging task.

Traditionally, it is thought that cases of unilateral breast reconstruction are more challenging from a symmetry perspective than bilateral cases. In an analysis by Losken et al. involving 1394 patients evaluating the frequency of symmetrizing procedures for unilateral breast reconstruction, 67% of delayed reconstruction patients and 22% of immediate cases underwent a contralateral symmetry procedure [1]. In their cohort, symmetry procedures were higher in implant-based reconstruction (89% delayed and 57% immediate) compared to autologous cases (59% delayed and 18% immediate) [1]. However, this study demonstrates that regardless of the timing or method of reconstruction, secondary procedures to achieve symmetry are key to achieving an optimal result. Though thought to be less challenging from a symmetry standpoint than unilateral reconstruction, bilateral breast reconstruction can also pose unique surgical challenges. For example, patients can have varying degrees of asymmetry preoperatively that make addressing each breast pocket individually critical. In addition, when a single side undergoes radiation therapy, the subsequent fibrosis and soft tissue changes can significantly alter symmetry between the breasts.

Various tools have been described to address postoperative asymmetry, including modifications to both the reconstructed or native breast, such as augmentation mammaplasty, reduction, mastopexy of the native breast, mastopexy of autologous flaps, and hybrid autologous/implant reconstruction. As a result, selecting which tool to use based on patient examination, reconstructive goals, and the reconstructive method requires experienced decision-making. This article, therefore, provides a review of the current literature, highlights case examples, and provides a treatment algorithm for obtaining symmetry.

## 2. Materials and Methods

**Figure 1:**
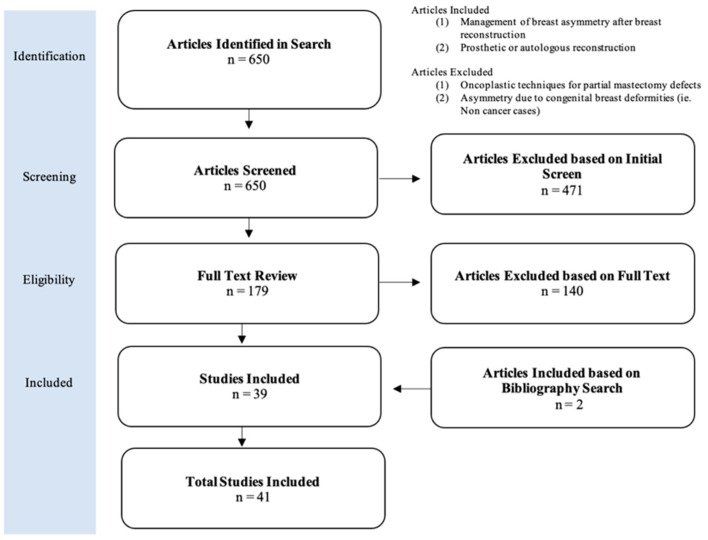
Preferred Reporting Items for Systematic Reviews diagram. This figure highlights the results of the systematic search according to PRISMA guidelines. Forty-one total studies met the inclusion criteria.

A systematic review was performed using the Preferred Reporting Items for Systematic Reviews and Meta-Analyses (PRISMA) statement guidelines (Figure 1). The search, including articles since inception through 2024, included the following bibliographic databases: PubMed, Cochrane Library, and Embase. The following search terms were used for PubMed and adapted as necessary for the additional databases to identify all possible papers that may meet this study’s inclusion criteria:

(“breast reconstruction” [Text Word] OR “alloplastic breast reconstruction” [Text Word] OR “autologous breast reconstruction” [Text Word] OR “implant-based breast reconstruction” [Text Word] OR “tissue expander” [Text Word]) AND (“symmetry” [Text Word] OR “asymmetry” [Text Word] OR “symmetrizing” [Text Word]).

Criteria for eligibility and exclusion were decided prior to performing the review. Patients undergoing either tissue-expander implant-based breast reconstruction or autologous reconstruction were included. Unilateral and bilateral breast reconstructions were considered, as were both immediate and delayed cases. Only studies that primarily focused on the management of asymmetry during the reconstructive process were included. Only studies focused on managing breast cancer patients, not benign breast disease (i.e., tuberous breast deformity, etc.) were considered. Oncoplastic techniques for partial mastectomy/lumpectomy defects were excluded. No exclusions were made based on the total number of patients or the follow-up period. Papers based solely on animal or in vitro models were excluded. Papers published in a language other than English were excluded. Bibliographies of all included articles were screened to identify additional articles not obtained in the preliminary search. The quality of all selected articles and risk of bias was assessed using the Methodological Index for Non-Randomized Studies (MINORS) score. Key findings and outcomes were reviewed for each included article, including type and timing of symmetrizing procedures performed, rate of complications, rate of revision, patient satisfaction, and measures of postoperative asymmetry, when provided.

## 3. Results

Searches of PubMed, Cochrane, and Embase databases yielded a total of six hundred and fifty unique articles based on our search criteria. Four hundred and seventy-one articles were removed for irrelevance based on titles and abstracts. One hundred and seventy-nine full-text articles were assessed for eligibility, after which thirty-nine articles were found to meet the predetermined inclusion criteria. After reviewing the bibliographies of the included articles, two additional studies met the inclusion criteria, totaling forty-one included articles (Figure 1).

All included studies were primary literature publications focused on management of asymmetry in cases of either implant-based or autologous breast reconstruction. Eighteen studies included only tissue-expander implant patients, sixteen studies included only autologous patients, and seven studies included a mix of both implant and autologous reconstruction. No randomized control trials were identified in the literature. Techniques used for management of asymmetry and outcome variables were too heterogenous to allow for meta-analysis. However, techniques and key outcomes were reviewed in the discussion pertaining to either mainly implant-based or autologous reconstruction. Table 1 provides the complete list of included studies.

## 4. Discussion

**Figure 2:**
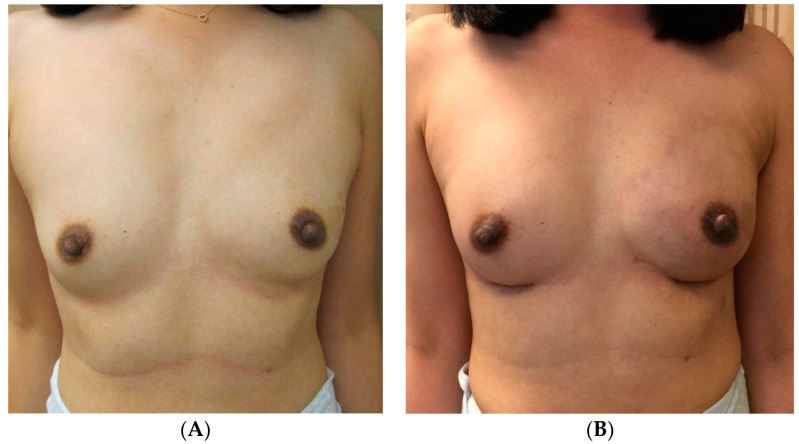
Unilateral implant reconstruction with contralateral augmentation. (**A**) The preoperative photos of a 32-year-old female who subsequently underwent left-breast tissue-expander implant reconstruction (submuscular, 375 mL silicone implant) and a right implant augmentation in the dual plane (255 mL silicone implant). (**B**) Postoperative results.

**Figure 3:**
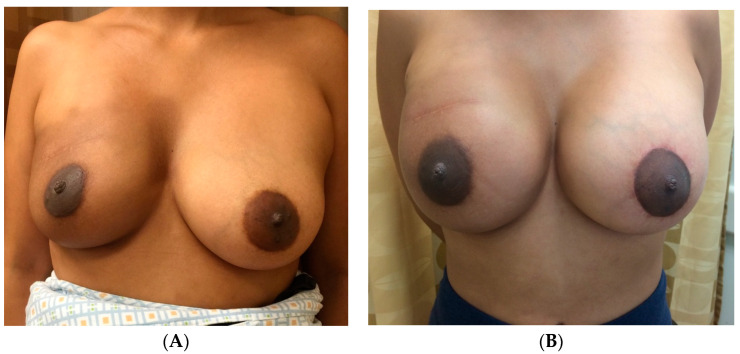
Unilateral implant reconstruction with prior radiation, contralateral augmentation mastopexy. (**A**) The preoperative photos of a 45-year-old female with a history of right breast conservation therapy with adjuvant radiation. She then underwent right-nipple-sparing mastectomy and eventual pre-pectoral implant reconstruction (495 mL silicone implant) with fat grafting (75 mL), with a contralateral symmetrizing augmentation (175 mL silicone implant) with circumareolar mastopexy. Postoperative results are shown in (**B**).

**Figure 4:**
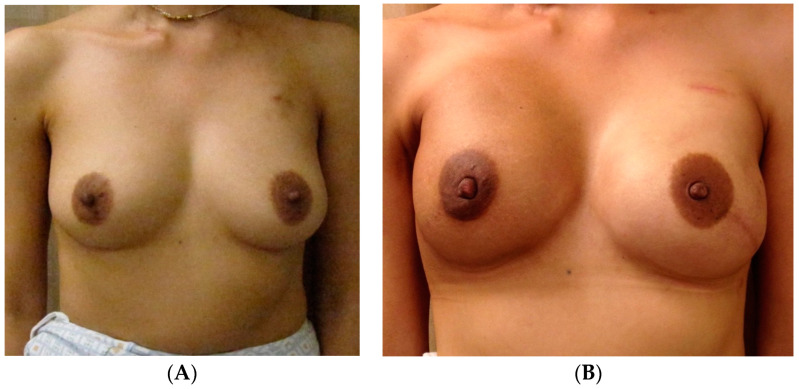
Bilateral implant reconstruction with radiation therapy. (**A**) Preoperative photos of a 53-year-old female with a history of Hodgkin’s lymphoma with mantle radiation. She was diagnosed with right breast cancer and underwent bilateral nipple-sparing mastectomy with immediate TE-implant reconstruction with adjuvant radiation to the right breast. (**B**) Her postoperative result, including a 475 mL right breast implant with 60 mL of fat grafting and a 425 mL left breast implant with 30 mL of fat grafting to achieve symmetry.

**Figure 5:**
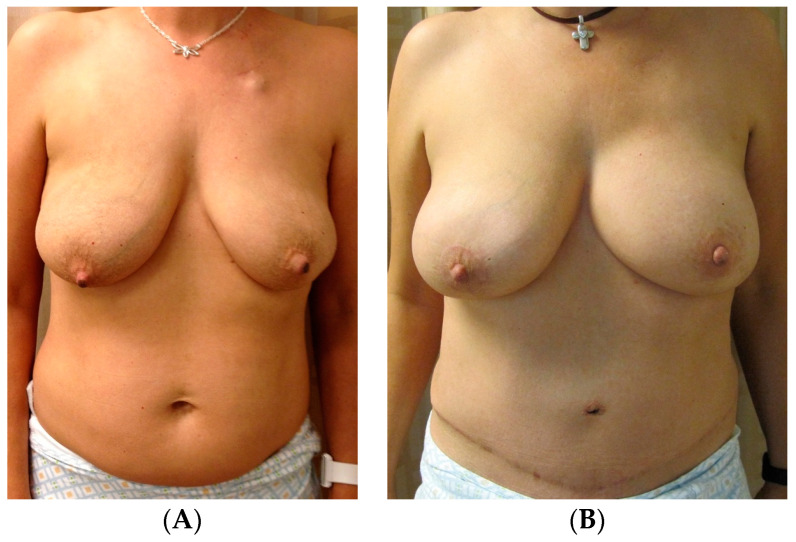
Unilateral autologous reconstruction with contralateral mastopexy. (**A**) The preoperative photos of a 49-year-old female who subsequently underwent a left-nipple-sparing mastectomy with immediate abdominal-based autologous reconstruction and a right vertical mastopexy for symmetry. She also underwent autologous fat grafting with 240 mL to the right and 120 mL to the left breast. Postoperative results are shown in (**B**).

**Figure 6:**
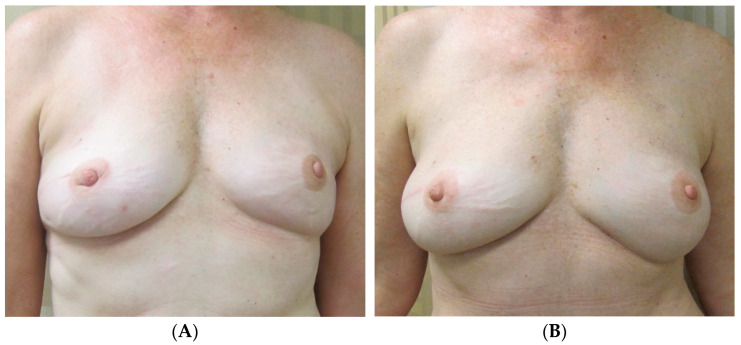
History of unilateral radiation, bilateral autologous reconstruction, and fat grafting. (**A**) The preoperative photos of a 55-year-old female with a history of left breast cancer and prior breast conservation therapy with unilateral radiation, highlighting the contracted and smaller appearance of the left breast. The patient then underwent bilateral nipple-sparing mastectomy with immediate abdominal free flaps with fat grafting to correct asymmetry, 90 mL to the right breast and 285 mL to the left breast. Postoperative results are shown in (**B**).

**Figure 7:**
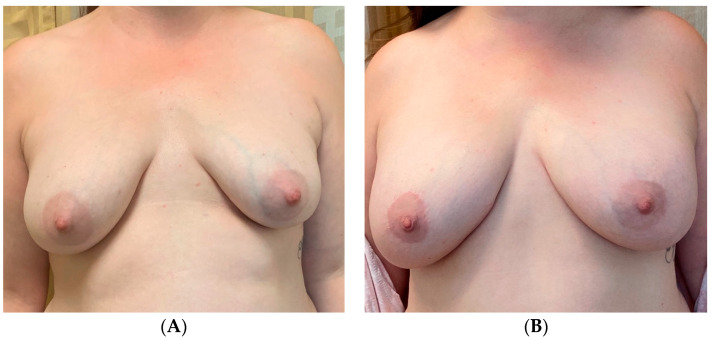
Bilateral autologous reconstruction with adjustable saline implant. (**A**) The preoperative photos of a 38-year-old female with a significant degree of baseline breast asymmetry who subsequently underwent bilateral nipple-sparing mastectomy with immediate bilateral abdominal free flaps with inflatable saline implants to adjust postoperative volume and improve final symmetry. Postoperative results are shown in (**B**).

**Figure 8:**
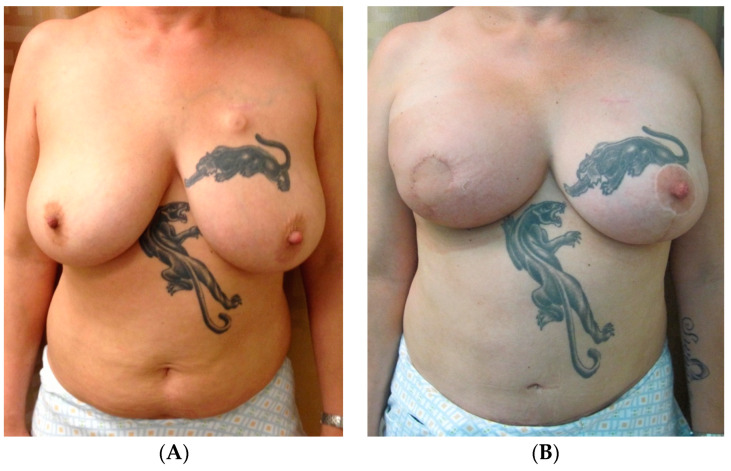
Unilateral autologous reconstruction with radiation, contralateral reduction. (**A**) The preoperative photos of a 43-year-old female diagnosed with right-sided breast cancer. She underwent right non-nipple-sparing mastectomy with immediate abdominal free flap reconstruction and postoperative radiation therapy. (**B**) Her final result after a symmetrizing left breast reduction with similar size and contour of the breasts.

The following discussion highlights the key findings and outcomes of the articles included in Table 1, as well as clinical cases from the senior author’s practice (Figure 2, Figure 3, Figure 4, Figure 5, Figure 6, Figure 7 and Figure 8) that relate to each section, starting with implant-based reconstruction in Section 4.1, followed by a focus on autologous reconstruction in Section 4.2.

### 4.1. Implant-Based Reconstruction

#### 4.1.1. Unilateral Implant Reconstruction

In cases of unilateral implant reconstruction, reconstructive surgeons face the challenge of creating a natural-appearing breast and achieving symmetry with the native breast. Losken et al. identified that 66% of patients who underwent unilateral implant-based reconstruction required a symmetry procedure, with augmentation being the most common (41%) [1]. Compared to the unilateral autologous group, these secondary symmetry procedures were significantly more common in implant-based reconstructions (66% versus 37%, *p* < 0.001) [1].

Several methods have been proposed to address asymmetry in unilateral implant reconstruction, most commonly focusing on the contralateral, native breast [2,3,4,5]. Typically, this involves implant augmentation of the contralateral side when the native breast is smaller or breast reduction/mastopexy when the native breast is larger and more ptotic [3,4,5,6]. In an analysis of 582 patients with unilateral implant reconstruction who underwent contralateral symmetry procedures performed by Barone et al., 235 patients (40%) underwent a contralateral implant-based procedure, 178 (31%) underwent contralateral breast reduction, and 169 (29%) underwent contralateral mastopexy [7]. By analysis of the Kroll scale, the implant-based contralateral procedure group received higher scores for final symmetry, shape, and overall aesthetic results compared to the reduction or mastopexy groups. The authors proposed that the contralateral implant is critical to fill the upper pole to match the reconstructed breast and to achieve a similar effect of aging on the two breasts [7]. Similarly, Razdan et al. reported that among their cohort of 553 patients with unilateral reconstruction and contralateral symmetry procedures, those with contralateral breast augmentation had greater satisfaction with the reconstructive outcome compared to controls and the reduction/mastopexy group [8].

Furthermore, other studies have highlighted nuanced techniques for optimal symmetry of the native breast, such as the concomitant crescent mastopexy with augmentation technique as well as the use of synthetic mesh as an inner bra sling in cases of symmetrization mastopexy to maintain symmetry long term [2,9]. Salgarello et al. proposed contralateral immediate symmetrization with a tailored reshaping of the native breast parenchyma with submuscular augmentation as a durable technique, focusing on removal of tissue at the base of the breast and lateral quadrants to better match the breast mound of the reconstructed side [10]. Figure 2 provides an example of contralateral augmentation for symmetry in unilateral implant reconstruction. This 32-year-old female with left-breast tissue-expander implant reconstruction (submuscular, 375 mL silicone implant) underwent a right implant augmentation in the dual plane (255 mL silicone implant) to achieve optimal postoperative symmetry between breasts. By contrast, in cases where the native breast was larger than the reconstructed breast, De Biasio et al. compared outcomes of simultaneous contralateral reduction mammaplasty with various glandular pedicles and noted improved outcomes with superior or medial pedicles compared to an inferior pedicle technique [11].

In addition to the variability in choosing the correct symmetry procedure, there are also multiple schools of thought regarding the timing of contralateral breast intervention. Most surgeons perform unilateral mastectomy with immediate breast reconstruction and adjust the contralateral breast to achieve symmetry at a second stage using the reconstructed breast as a template, but there is also growing evidence in support of simultaneous symmetry procedures to the contralateral breast to minimize the total number of operations [12,13]. Rancati et al. compared outcomes of patients undergoing simultaneous contralateral symmetrization versus delayed contralateral symmetrization and noted that the delayed cohort required more procedures (3.4 vs. 1.8, *p* < 0.0001) but a shorter hospitalization length (2.8 vs. 4.1 days, *p* < 0.0001) [12]. Similarly, Smith et al. advocated that balancing the procedures at the time of breast reconstruction is safe and effective, even more so in autologous reconstructions, where 87% of their patients did not require a second operation [14].

Achieving symmetry between the breasts becomes even more challenging in the setting of unilateral radiation on the reconstructed side. It is well known that adjuvant radiation therapy increases surgical complications, particularly in tissue-expander implant-based breast reconstruction [42,43]. Traditionally, to mitigate the risk of complications, including implant exposure or capsular contracture for patients who anticipate adjuvant radiotherapy, devices are placed in the submuscular or dual plane [42,44]. However, more recent data propose that outcomes in patients undergoing radiation after pre-pectoral device placement may be acceptable, suggesting that radiation may not be an absolute contraindication for pre-pectoral reconstruction [42,44]. These considerations for pocket placement are critical when considering implant augmentation on the contralateral native breast. If the reconstructed pocket is pre-pectoral, the augmentation side is typically sub-glandular in our practice. If the reconstructed side is submuscular, the augmentation side is placed in the dual plane to provide optimal symmetry. A total submuscular implant placement on the native side is avoided to prevent the development of a “double-bubble” effect over time as the breast tissue drifts over the implant.

Especially in the setting of adjuvant radiation therapy, autologous fat grafting has become a powerful tool for improving both the soft tissue envelope and addressing contour deformities [45,46]. Most commonly, fat grafting is implemented after implant placement to address superior pole rippling or chest wall step-offs [15]. Fat grafting can also be performed while the tissue expander is in place for those with thin mastectomy flaps or those undergoing radiation therapy in hopes that the regenerative potential of the fat improves soft tissue viability and mitigates fibrotic changes in radiated regions. In addition, autologous fat grafting can be used to augment the contralateral native breast and has even been used in conjunction with the Brava device, a vacuum-based external soft-tissue-expansion system, to increased fat viability [16].

Figure 3 highlights a 45-year-old female with a history of right breast conservation therapy with adjuvant radiation who then underwent right-nipple-sparing mastectomy and pre-pectoral implant reconstruction (495 mL silicone implant) with fat grafting (75 mL), with a contralateral symmetrizing augmentation (175 mL silicone implant) circumareolar mastopexy. Postoperative results shown in Figure 3B demonstrate that the combination of fat grafting to soften the right breast skin envelope on the radiated side and augmentation mastopexy on the contralateral breast to match upper pole projection and nipple position achieved an optimal, symmetric result.

More recently, decellularized adipose matrices (DAMs) have also been introduced to treat both contour irregularities and radiation-induced skin fibrosis, similar to autologous fat grafting [47]. For example, Renuva (MTF biologics, Edison, NJ, USA) provides an adipogenic extracellular matrix scaffold that has been used for breast contour deformities with early success; however, long-term data are necessary to compare efficacy to autologous fat [47].

#### 4.1.2. Bilateral Implant Reconstruction

Though achieving symmetry in bilateral implant reconstruction may be more straightforward than in unilateral cases with a native ptotic breast, asymmetry between the implant pockets or unilateral radiation can lead to unique challenges in this patient population as well [1,17]. For example, many women demonstrate preoperative asymmetry both in breast size and shape [48]. If the degree of asymmetry is significant, patients may require a unilateral mastopexy to tighten the skin envelope and lift the breast to achieve a symmetric result after reconstruction with an implant. Alternatively, shape modifications can be made by adjusting the implant pocket, such as using capsulorrhaphy techniques, if necessary. In a study by Nores et al., the authors noted improvement in nipple position asymmetry after TE to the implant second stage, likely due to the aforementioned pocket alteration techniques available [17]. Mercury et al. similarly noted that patients with severe asymmetry at baseline who underwent bilateral two-stage TE to implant reconstruction could achieve improved symmetry of the nipple positions compared to those who underwent direct to implant reconstruction, suggesting that intraoperative positioning of the nipples over the TE with adherent dressings may be beneficial [18]. However, if nipple asymmetry persists or if there is a significant degree of ptosis after bilateral reconstruction, Wise pattern mastopexy using bi-pedicled dermal-fat flaps has been described [19]. Salibian et al. [19] emphasized several key points for performing secondary mastopexy safely in this population: thick mastectomy skin flaps, a waiting period of 3–4 months after reconstruction to allow the capsule to mature and collateral circulation to the nipple/areola to improve, and avoiding disturbing the capsule if possible (if the implant is exchanged, a single vertical incision along the margin of the inferior dermal-fat pedicle is advised).

Asymmetry between breasts, particularly in cases of nipple-sparing mastectomy, can be further exaggerated in cases of unilateral radiation to the oncologic side [17,18]. In an analysis by Small et al., nipple malposition occurred in 13% of 319 nipple-sparing mastectomies with immediate device-based reconstruction, with prior radiation as a significant risk factor [20]. Surgical techniques for correction in their series included crescent mastopexy, implant exchange and pocket revision, conversion to free nipple grafts, and pedicled nipple transposition. Even in non-nipple-sparing cases, radiation-induced fibrosis to the skin envelope can result in asymmetry between the breasts. In order to mitigate radiation-induced asymmetry from capsular contracture and superior migration of the irradiated reconstructed side, Roh et al. proposed a differential acellular dermal matrix (ADM) inset in cases of bilateral reconstruction with planned unilateral adjuvant radiation [21]. The authors suggested that setting the ADM lower and overexpanding the volume on the side requiring radiation, compared to the non-radiated side, resulted in improved symmetry, as the radiated side migrated superiorly over time [21]. Minor differences in shape and contour can often be addressed with autologous fat-grafting techniques, but significant differences after unilateral radiation may require more extensive pocket revision or implant exchange to either breast, depending on patient preference.

Figure 4A demonstrates preoperative photos of a 53-year-old female with a history of Hodgkin’s lymphoma with mantle radiation. She was diagnosed with right breast cancer and underwent bilateral nipple-sparing mastectomy with immediate TE-implant reconstruction with adjuvant radiation to the right breast. To achieve symmetry, a larger implant and higher volume of fat grafting were used on the radiated breast. Figure 4B demonstrates her postoperative result, including a 475 mL right breast implant with 60 mL of fat grafting and a 425 mL left breast implant with 30 mL of fat grafting, with appropriate symmetry.

### 4.2. Autologous Reconstruction

#### 4.2.1. Unilateral Autologous Reconstruction

Unilateral autologous reconstruction is thought to have a higher probability of postoperative asymmetry compared to bilateral reconstruction [1]. However, the need for secondary symmetrizing procedures is a complex decision that incorporates patient preferences and post-reconstruction goals, the type of mastectomy planned, pre-mastectomy breast characteristics, and which reconstructive method is being used. In a review of 1394 patients, 37% of patients who underwent unilateral autologous reconstruction underwent a symmetry procedure, with reduction mammaplasty being the most common [1]. Similarly, Giacolone et al. reported that contralateral symmetry procedures were performed in 34% of patients who underwent unilateral transverse rectus abdominis musculocutaneous reconstruction [22]. Similar to prosthetic reconstruction, there is also ongoing debate regarding optimal timing of symmetrizing procedures in the autologous realm, with a growing body of evidence supporting immediate symmetrization [23,24,25,26,27,28]. For example, Huang et al. reported increased patient satisfaction, minimally increased operative times, and no increased complication profile for immediate contralateral reduction/mastopexy for unilateral autologous reconstruction [29]. In a study by Chang et al., immediate contralateral procedures had a higher complication rate than delayed (9.7% vs. 4.0%, *p* = 0.01), but the total number of procedures per patient was significantly reduced (1.84 vs. 2.45, *p* < 0.05). In addition, contralateral immediate breast reductions had a lower rate of revision than immediate contralateral augmentation or mastopexy, emphasizing to surgeons that the risk of revision depends on the specifics of the symmetrizing procedure chosen [30].

The above strategies focus primarily on the contralateral native breast, but in some cases, it is more appropriate to focus on the reconstructed breast itself. In contrast to Losken et al. and Giacalone et al., Nahabedian et al. performed ipsilateral symmetry procedures four times more often than contralateral procedures [31]. Recontouring of the skin and fat by direct excision was the most common revision procedure, followed by reduction mammaplasty, mastopexy, and implant augmentation. In this study of 382 autologous reconstruction patients, 10.5% of patients had contralateral procedures, compared to 42% for the ipsilateral breast [31]. However, this study did not specifically detail the recontouring techniques used for the flap or describe variations in the timing of the intervention. In a follow-up study, Nahabedian hypothesized that this discrepancy among authors could be due to the fact that the Losken autologous reconstruction group was composed mostly of patients with pedicled TRAM flaps, compared to mostly free tissue transfer in the first Nahabedian study [32]. This highlights a critical point that the specific mechanism of autologous reconstruction likely impacts the incidence of symmetrization procedures and best practices for management.

Troy et al. performed a retrospective review of all patients at a single institution over a seven-year period who underwent flap mastopexy on one or both breasts after autologous reconstruction [33]. In this study, the authors performed an inverted diamond mastopexy, which included a full-thickness excision inferiorly of the flap down to the chest wall, with more tissue removed from the IMF to improve the shape and projection. None of the patients in this series experienced flap loss, fat necrosis, or nipple loss after flap mastopexy. As opposed to standard mastopexy on the native breast, flap mastopexy requires understanding of flap vascularity in order to prevent compromising long-term flap survival. Troy et al. described successfully performing this technique as early as three months after reconstruction, such that it can be performed at the time of nipple reconstruction [33]. Similarly, Zafar et al. waited at least three months prior to revision to allow the reconstructed breast to “settle” and allow for neovascularization [34]. In our practice, particularly in the case of nipple-sparing mastectomy with autologous reconstruction, we favor waiting a minimum of six months (preferably at least one year) prior to offering mastopexy or reduction of the flap side to optimize revascularization of the nipple–areola complex. In this scenario, ICG-SPY is also a helpful adjunct to assess perfusion. In contrast, some patients may want to create a reconstructed breast that matches the natural ptosis of their native breast rather than undergoing flap mastopexy. Nanhekhan et al. proposed a method of creating a more ptotic reconstructed breast in the setting of delayed autologous reconstruction by dividing the native breast skin below the mastectomy scar into two dermocutaneous triangular flaps that are turned outward to create more ptosis of the flap [35]. This method can be particularly useful in those who have limited donor site tissue. In unilateral cases, utilization of a bi-pedicled abdominal flap has also shown to provide natural-appearing ptosis and projection [36].

An additional, versatile tool for addressing asymmetry in almost any type of breast reconstruction, including cases of unilateral autologous reconstruction, is fat grafting. For instance, autologous fat may be used to improve the volume in patients who desire a larger reconstructed side to match the native breast, or vice versa. This may be particularly useful for thin patients undergoing autologous reconstruction, who may otherwise consider prosthetic devices for volume augmentation [45,49,50]. Figure 5 highlights a 49-year-old female who underwent left-nipple-sparing mastectomy with immediate abdominal-based autologous reconstruction and a right vertical mastopexy for symmetry. To achieve symmetry of volume and shape, she also underwent autologous fat grafting with 240 mL to the right and 120 mL to the left breast, with the postoperative result shown in Figure 5B.

Beyond addressing volume discrepancies between the reconstructed and native breast, fat grafting also addresses a wide variety of contour deformities. For example, fat grafting can be used to smooth the transition between the autologous flap and native chest wall to prevent visible step-offs. Removal of costochondral cartilage for exposure of internal mammary vessels can also exacerbate upper pole concavities that can be addressed with autologous fat [45,49]. In addition to volume and contour, injection of autologous fat in regions of prior fat necrosis has been shown to assist in breakdown of fibrosis, facilitation of tissue remodeling, and improvements in local vascularity [45,51].

Though fat grafting is often used to augment the volume of the reconstructed breast, some patients have insufficient autologous fat to achieve their goal volumes or have a significant discrepancy in the size of the autologous donor tissue compared to their native breast, such that alternative options need to be considered. In this subset of patients, a hybrid approach to reconstruction using both an implant and autologous tissue can be used [37,38,39,40]. In a systematic review and pooled analysis performed by Piper et al., it was demonstrated that this hybrid technique can be performed with comparable, and perhaps even lower, complication rates than either technique alone [52]. In this cohort of 378 flaps, the implants were most frequently placed at the immediate time of autologous reconstruction (67%) and in the subpectoral plane (67%). There were no cases of implant migration causing compromise to the pedicle. Overall, flap loss rates were comparable to standard autologous reconstruction, and the implant loss rate was lower than patients with implant-only reconstruction, likely due to the overlying vascularized autologous tissue [52]. This suggests that implant augmentation of autologous tissue is not only safe but also expands the number of patients who are candidates for unilateral autologous reconstruction.

#### 4.2.2. Bilateral Autologous Reconstruction

Several of the above-mentioned methods remain applicable to patients undergoing bilateral autologous reconstruction. In bilateral autologous reconstruction for relatively thin patients, the use of autologous fat may help patients achieve a larger total volume reconstruction postoperatively to meet their reconstructive goals. In addition, the two sides of autologous reconstruction are inherently not identical—this can be due to slight variations in the donor site on each side, changes in vascularity that result in regions of fat necrosis on one side, or preoperative breast asymmetries that remain after reconstruction. Thus, fat grafting can be a useful tool for managing subtle differences between the two breasts. Figure 6 demonstrates a challenging case of addressing preoperative asymmetry with autologous reconstruction and fat grafting. The patient is a 55-year-old female with a history of left breast cancer and prior breast conservation therapy with unilateral radiation, resulting in a contracted and smaller appearance of the left breast. The patient then underwent bilateral nipple-sparing mastectomy with immediate abdominal free flaps with fat grafting to correct asymmetry, 90 mL to the right breast and 285 mL to the left breast. The postoperative results shown in Figure 6B show appropriate matches in contour, volume, and nipple position.

As previously mentioned for unilateral asymmetry, adding implants for cases of bilateral autologous reconstruction can also be a useful tool. In a review by Piper et al., the implant size ranged from 90 to 510 cc, with the most common sizes in the 200 cc range [52]. If a patient were to have a significant volume discrepancy between sides in bilateral cases, the size of the implants could be adjusted accordingly. In addition, adjustable saline implants can be used in combination with autologous reconstruction, such that the volume can be adjusted even after the procedure is complete. For example, Figure 7 highlights a case of a 38-year-old female with a significant degree of baseline breast asymmetry who subsequently underwent bilateral nipple-sparing mastectomy with immediate bilateral abdominal free flaps with inflatable saline implants to adjust the postoperative volume and improve the final symmetry.

Aside from volume, patients may also have a significant degree of asymmetry in the shape and degree of ptosis of their breasts. Cheong et al. performed a statistical analysis of natural breast symmetry in preoperative breast cancer patients and noted that a substantial portion of women (41.4%) showed sternal notch to nipple distance differences > 5 mm between their right and left breasts [48]. Specifically in cases of nipple-sparing mastectomy, this poses an interesting challenge to the reconstructive surgeon to achieve a symmetric result postoperatively. Such asymmetry can be corrected with a lift or reduction on one or both sides, if desired. Similar to what was previously discussed in unilateral scenarios, in our practice, we favor waiting a minimum of six months (preferably at least one year) prior to offering mastopexy or reduction of a flap reconstruction to optimize revascularization of the nipple–areola complex.

#### 4.2.3. Radiation in the Setting of Autologous Reconstruction

Postmastectomy radiation therapy is often necessary for patients with nodal spread or large primary tumors, but it can negatively impact both implant-based and autologous breast reconstruction outcomes. The effect of radiation on tissues is well documented and includes fibrosis and contracture, which often result in asymmetry. However, data regarding optimal timing of autologous reconstruction in patients who require postmastectomy radiation therapy remain controversial. In a study by Rogers et al. that utilized a matched-pairs analysis to investigate the impact of radiation on autologous tissue, the incidences of fat necrosis, fibrosis, and flap contracture were all significantly higher in the irradiated group, favoring a delay in autologous reconstruction until radiation is complete [53]. These results have been supported by additional studies that suggest poorer aesthetic outcomes, increased contracture, and increased hyperpigmentation when radiating the flap [54,55].

In contrast, Clarke-Pearson et al. analyzed a subset of patients who underwent immediate bilateral abdominal free flap reconstruction and subsequently underwent unilateral adjuvant radiation [56]. In their cohort, there was no clinically significant difference in rates of fat necrosis or breast shape distortion after radiation. The authors offered several techniques to optimize aesthetic outcomes when radiating the autologous tissue. To optimize flap vascularity and decrease the risk of fat necrosis, all patients in their study underwent preoperative magnetic resonance angiography to identify dominant perforators from the deep inferior epigastric system. The authors also argued that dead-space cavities result in uncontrolled contracture that result in shape distortion after radiation; therefore, each flap was secured around its periphery with sutures, and the mastectomy pocket was closed down laterally to eliminate all dead space. As a result, they proposed that with a meticulous technique, radiation should not preclude immediate autologous reconstruction [56].

When unilateral radiation in the autologous setting does result in asymmetry, a majority of the tools previously described can be utilized depending on patients’ needs. This includes fat grafting, which can be used to soften the skin envelope of the radiated side, improve contour deformities, or increase the overall breast volume. However, patients with prior radiation therapy often require more rounds of autologous fat grafting compared to non-radiated patients [41]. If the non-radiated breast is more ptotic or has a less projected upper pole, one could consider contralateral mastopexy or implant augmentation, respectively. Figure 8 highlights the case of a 43-year-old female diagnosed with right-sided breast cancer who underwent a right non-nipple-sparing mastectomy with immediate abdominal free flap reconstruction and postoperative radiation therapy to the flap. Figure 8B demonstrates her final result after a symmetrizing left-sided breast reduction, with a similar size and contour of the breasts postoperatively.

A critical compromise regarding the timing of radiation was presented in the literature when Kronowitz et al. introduced the concept of the delayed–immediate technique in 2004 [57]. By placing a tissue expander at the time of mastectomy, the delayed–immediate approach preserves the skin envelope while avoiding radiation to the autologous reconstruction. In our clinical practice, when patients have a high likelihood of requiring adjuvant radiation, we favor radiating prior to the final flap reconstruction using this delayed–immediate technique. As described by Zafar et al., this gives us the opportunity to replace radiated mastectomy skin flaps, particularly in the inferior and lateral subunits, if needed with a flap skin paddle to create natural ptosis [34].

The previously mentioned hybrid technique is also a possibility uniquely equipped to address the asymmetry that accompanies radiation. In the cohort previously described by Piper et al., 17.5% of patients had pre-reconstruction radiation and 8.2% underwent post-reconstruction radiation [52]. It is thought that the increased vascularity and robust soft tissue coverage of the autologous tissue over the implant provides protection in the setting of radiation, mitigating the degree of fibrosis [52]. In addition, hybrid reconstruction with the use of inflatable saline implants could also allow for outpatient adjustment of the volume to adjust asymmetry that develops between the breasts after radiation. Zhou et al. reported high patient satisfaction with immediate placement of pre-pectoral adjustable saline implants with abdominal free tissue transfer, emphasizing the advantage of being able to adjust the breast volume post-reconstruction with this technique, particularly with patients who undergo adjuvant radiation [58].

### 4.3. Treatment Algorithm

**Figure 9:**
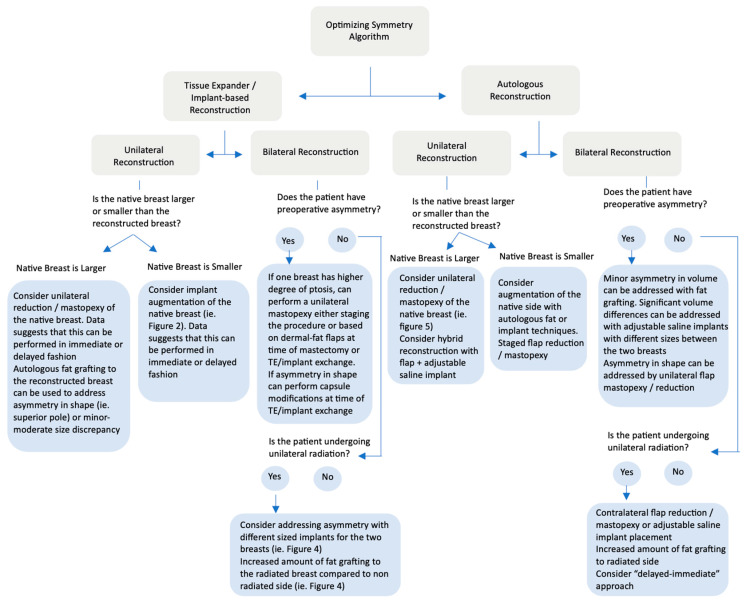
Algorithm for optimizing symmetry in breast reconstruction. This treatment algorithm provides recommendations for obtaining symmetry in cases of both unilateral and bilateral prosthetic and autologous reconstruction techniques.

Based on the previous discussion of the included articles in the systematic review, as well as the authors’ clinical experience highlighted in the prior case examples, a treatment algorithm for addressing asymmetry in breast reconstruction was then developed by the authors and is shown in Figure 9.

## 5. Conclusions

As a result of the advances in oncologic surgery and breast reconstruction techniques, patients and surgeons continue to raise the bar for the aesthetic quality of breast reconstruction outcomes. Whether reconstruction is unilateral or bilateral, delayed or immediate, implant-based or autologous, achieving symmetry between the breasts can be challenging. Various techniques exist to achieve improved symmetry, typically in the form of secondary revision procedures, but choosing which tools to use, addressing the native or contralateral breast, and the timing of secondary procedures remains a challenging topic. As a result, this review aims to serve as a comprehensive decision-making guide for both patients and surgeons. Limitations of this study include the lack of randomized controlled trials available for the study population and a vast majority of retrospectively collected data. In addition, the variable measures of pre- and post-operative asymmetry made meta-analysis challenging. Future studies with standardized measures of symmetry with prospectively collected data are necessary.

## Figures and Tables

**Table 1:** Summary of included articles. TE: tissue expander, XRT: radiation therapy, DTI: direct to implant, NSM: nipple-sparing mastectomy, and DIEP: deep inferior epigastric perforator flap.

Reference Number	Author	Year of Publication	MINORS Score	Type of Reconstruction	Size of Patient Population	Level of Evidence	Key Findings
[1]	Losken	2002	18	Autologous and implant-based reconstruction	1394	III	• Trends in the management of unilateral breast cancer from delayed to immediate reconstruction and from implants to autologous tissue have reduced the incidence of contralateral symmetry procedures • Reduction mammaplasty is the most common symmetry procedure used for autologous tissue reconstruction, augmentation predominates when implants are used
[2]	Cogliandro	2018	15	Implant-based reconstruction	55	III	• For mild ptosis of the contralateral breast, crescent mastopexy provides improved symmetry with minimal scars
[3]	Kropf	2011	8	Implant-based reconstruction	26	IV	• After unilateral TE/implant reconstruction, combined trans-axillary augmentation/ peri areolar mastopexy of the contralateral breast is a reliable way to improve symmetry for grade I or II ptosis
[4]	Ho	2017	10	Implant-based reconstruction	71	III	• Contralateral augmentation trans midline scarless (CATS) technique to simultaneously augment the contralateral breast can be performed safely and improve symmetry after unilateral reconstruction
[5]	Serra	2008	9	Implant-based reconstruction	50	IV	• “Elliott’s technique”—double superior pole and vertical inferior pole plication—is a suitable and long-lasting procedure for small-moderate ptotic breast and elastic skin to achieve contralateral symmetry after unilateral reconstruction
[6]	Kim	2017	15	Implant-based reconstruction	52	IV	• Contralateral breast augmentation at the time of TE to Implant exchange can improve patient satisfaction and symmetry
[7]	Barone	2018	12	Implant-based reconstruction	582	IV	• Patients with unilateral implant based breast reconstruction who underwent contralateral implant augmentation had better BREAST-Q scores for symmetry than those who underwent contralateral reduction or mastopexy
[8]	Razdan	2019	9	Implant-based reconstruction	553	III	• Prosthetic reconstruction with contralateral breast augmentation was associated with greater satisfaction with reconstructive outcome • breast reduction and mastopexy procedures demonstrated equivalent satisfaction with breasts compared with controls but may be associated with lower physical well-being
[9]	Jozsef	2020	20	Implant-based reconstruction	117	IV	• ULTRAPRO mesh sling symmetrization can be successfully used to decrease pseudoptosis and nipple down-migration, offering a safe alternative for long-lasting symmetry and high patient satisfaction
[10]	Salgarello	2015	10	Implant-based reconstruction	19	IV	• Simultaneous contralateral symmetrization after unilateral immediate implant reconstruction after NSM facilitates durable symmetric outcomes.
[11]	Biasio	2017	18	Implant-based reconstruction	90	IV	• Superior or medial pedicle reduction mammaplasties seem to better preserve breast shape and position, and they maintain a more similar appearance to the contralateral prosthetic breast over time
[12]	Rancati	2022	18	Implant-based reconstruction	105	IV	• Immediate contralateral symmetry procedures can be performed safely and reliably
[13]	Qiu	2023	10	Implant-based reconstruction	33	IV	• Direct-to-implant breast reconstruction and simultaneous contralateral endoscopic augmentation is an ideal alternative for patients with small breasts for improving symmetry and cosmetic results
[14]	Smith	2014	18	Autologous and implant-based reconstruction	102	IV	• Performing balancing at the time of breast reconstruction is safe and most effective in autologous reconstructions, where 87% did not require a second operation for symmetry.
[15]	Cogliandro	2017	20	Implant-based reconstruction	70	III	• Autologous fat grafting can be used to improve cosmetic outcomes and reduce postoperative pain after implant-based breast reconstruction.
[16]	Uda	2015	10	Autologous and implant-based reconstruction	12	IV	• Shaping the unaffected breast by Brava-assisted autologous fat grafting is predictable, effective, and feasible as an aesthetic adjunct to unilateral breast reconstruction to achieve breast symmetry
[17]	Nores	2022	10	Implant-based reconstruction	23	IV	• TE nipple asymmetry predicts final nipple symmetry • TE to implant exchange can improve asymmetry with pocket manipulation • XRT worsens nipple symmetry after final stage
[18]	Mercury	2022	17	Implant-based reconstruction	68	IV	• Both pre pectoral and subpectoral implant placement in DTI patients resulted in increased asymmetry post op, but was significant for prepectoral group
[19]	Salibian	2018	10	Implant-based reconstruction	155	IV	• Mastopexy after TE-Implant reconstruction can be safely performed to correct ptosis and improve breast shape and symmetry
[20]	Small	2014	11	Implant-based reconstruction	319	III	• NSM followed by immediate device-based reconstruction has a significant risk of nipple malposition, occurring in 14% of this cohort of 319 patients.
[21]	Roh	2017	8	Implant-based reconstruction	25	IV	• A differential ADM inset and TE pocket creation for bilateral TE/ADM breast reconstructions with planned unilateral PMRT can minimize the typical adverse aesthetic effects of PMRT on reconstruction results and maximize symmetry
[22]	Giacalone	2002	17	Autologous and implant-based reconstruction	683	III	• TRAM flap breast reconstruction more frequently matches the opposite breast, thus avoiding additional surgery to achieve symmetry in comparison with implant-based techniques
[23]	Stevenson	1993	17	Autologous reconstruction	128	III	• Performing single stage TRAM flap reconstruction with contralateral reduction or mastopexy is safe and yields a satisfactory aesthetic result
[24]	Giordano	2019	18	Autologous reconstruction	78	III	• Immediate symmetrization is feasible in autologous LD breast reconstructions • 76% of patients did not require a second operation for symmetry
[25]	Inbal	2012	17	Autologous reconstruction	51	IV	• Simultaneous contralateral symmetry procedures in unilateral DIEP breast reconstruction is safe and effective for cosmetic outcomes
[26]	Chang	2013	10	Autologous reconstruction	77	III	• For patients who need contralateral reduction mammoplasty or mastopexy for symmetry, performing these procedures and breast reconstruction simultaneously facilitates single-stage breast reconstruction in most patients
[27]	Ulusal	2006	11	Autologous reconstruction	158	III	• Simultaneous contralateral breast augmentation with DIEP or superficial inferior epigastric artery flap surgery can be performed with high success rates and poses low surgical risk/morbidity
[28]	Laporta	2016	18	Autologous reconstruction	48	III	• Simultaneous contralateral balancing procedures in unilateral DIEP flap reconstruction using a symmetrization algorithm results in comparable aesthetic outcomes and patient satisfaction to a staged procedure
[29]	Huang	2011	18	Autologous reconstruction	22	III	• Simultaneous contralateral balancing procedures including reduction/mastopexy in selected patients can be performed with unilateral breast reconstruction using free abdominal flaps with greater patient satisfaction, minimal increase in operative time, and no increase in complication rates
[30]	Chang	2015	18	Autologous reconstruction	1120	III	• Half of patients undergoing a unilateral flap for breast reconstruction also underwent a contralateral balancing procedure • Immediate contralateral augmentation and mastopexy carry a higher revision rate, consider performing them in a staged fashion • No differences in rate of revisions for breast reductions, therefore simultaneous contralateral reduction is reasonable
[31]	Nahabedian	2005	16	Autologous and implant-based reconstruction	382	III	• Initial volume symmetry was observed more often following autologous reconstruction, whereas initial contour symmetry was obtained more often with implant reconstruction • Final volume and final contour symmetry were obtained more often with autologous tissue reconstruction. Secondary procedures were performed more often following autologous reconstruction.
[32]	Nahabedian	2008	18	Autologous and implant-based reconstruction	945	III	• Ipsilateral procedures are more common than contralateral procedures in the setting of autologous reconstruction, delayed reconstruction, and immediate reconstruction, but are nearly equivalent in the setting of prosthetic reconstruction
[33]	Troy	2018	9	Autologous reconstruction	10	IV	• Flap mastopexy provides a reliable method to adjust the inframammary fold, increase projection, and address excess ptosis • It can be performed as early as 3 months after initial reconstruction
[34]	Zafar	2015	9	Autologous reconstruction	6	V	• Performing reduction and mastopexy of DIEP flap breast reconstruction is challenging, and requires consideration of neovascularization to the nipple areolar complex, consideration of prior scars when planning skin resection pattern, and special considerations for reshaping the parenchyma
[35]	Nanhekhan	2012	6	Autologous reconstruction	43	IV	• The authors present their method of shaping a ptotic breast after delayed autologous breast reconstruction, which includes dividing the native breast skin below the mastectomy scar into two dermo cutaneous triangular flaps
[36]	Wang	2015	10	Autologous reconstruction	126	IV	• For patients with a relatively tight abdomen undergoing autologous breast reconstruction, specific abdominal flap design and shaping approaches can improve symmetry postoperatively
[37]	Alhefzi	2020	10	Autologous reconstruction	24	III	• Secondary augmentation of abdominal-based flaps using an implant is an effective method to address volume and asymmetry • However, postoperative surgical site infection occurred in 17% of patients.
[38]	Bach	2020	9	Autologous reconstruction	13	IV	• “Hybrid breast reconstruction” using an implant underneath a free flap provided a safe and reliable option to optimize breast reconstruction outcomes
[39]	Figus	2009	12	Autologous reconstruction	156	III	• Both primary and delayed DIEP/implant augmentation can be a safe and effective option in optimizing breast reconstruction with autologous tissue
[40]	Walters	2015	9	Autologous reconstruction	7	IV	• Delayed augmentation mammoplasty after DIEP flap breast reconstruction is a safe and feasible procedure for patients who lack adequate abdominal tissue
[41]	Losken	2011	18	Autologous and implant-based reconstruction	107	IV	• Autologous fat grafting is a safe and effective tool for secondary breast reconstruction. It is helpful in all types of reconstructions to improve contour, volume, and overall breast shape and symmetry • Repeat injections are often required and this is more common in patients with longer follow-up and in those with a history of radiation therapy

## Data Availability

The original contributions presented in the study are included in the article, further inquiries can be directed to the corresponding author.
